# Three-dimensional identification of microvascular pathology and neurovascular inflammation in severe white matter hyperintensity: a case report

**DOI:** 10.1038/s41598-024-55733-y

**Published:** 2024-02-29

**Authors:** Gemma Solé-Guardia, Matthijs Luijten, Bram Geenen, Jurgen A. H. R. Claassen, Geert Litjens, Frank-Erik de Leeuw, Maximilian Wiesmann, Amanda J. Kiliaan

**Affiliations:** 1 Department of Medical Imaging, Anatomy, Donders Institute for Brain, Cognition & Behavior, Preclinical Imaging Center PRIME, Radboud Alzheimer Center, Radboud university medical center, 6525 EZ Nijmegen, PO Box 9101, The Netherlands; 2 Department of Geriatrics, Donders Institute for Brain, Cognition & Behavior, Radboud Alzheimer Center, Radboud university medical center, Nijmegen, The Netherlands; 3 Department of Cardiovascular Sciences, University of Leicester, Leicester, UK; 4 Department of Pathology, Radboud university medical center, Nijmegen, The Netherlands; 5 Computational Pathology Group, Research Institute for Medical Innovation, Radboud university medical center, Nijmegen, The Netherlands; 6 Department of Neurology, Donders Institute for Brain, Cognition & Behavior, Radboud university medical center, Nijmegen, The Netherlands

**Keywords:** Cerebrovascular disorders, White matter disease, Light-sheet microscopy

## Abstract

White matter hyperintensities (WMH) are the most prevalent markers of cerebral small vessel disease (SVD), which is the major vascular risk factor for dementia. Microvascular pathology and neuroinflammation are suggested to drive the transition from normal-appearing white matter (NAWM) to WMH, particularly in individuals with hypertension. However, current imaging techniques cannot capture ongoing NAWM changes. The transition from NAWM into WMH is a continuous process, yet white matter lesions are often examined dichotomously, which may explain their underlying heterogeneity. Therefore, we examined microvascular and neurovascular inflammation pathology in NAWM and severe WMH three-dimensionally, along with gradual magnetic resonance imaging (MRI) fluid-attenuated inversion recovery (FLAIR) signal (sub-)segmentation. In WMH, the vascular network exhibited reduced length and complexity compared to NAWM. Neuroinflammation was more severe in WMH. Vascular inflammation was more pronounced in NAWM, suggesting its potential significance in converting NAWM into WMH. Moreover, the (sub-)segmentation of FLAIR signal displayed varying degrees of vascular pathology, particularly within WMH regions. These findings highlight the intricate interplay between microvascular pathology and neuroinflammation in the transition from NAWM to WMH. Further examination of neurovascular inflammation across MRI-visible alterations could aid deepening our understanding on WMH conversion, and therewith how to improve the prognosis of SVD.

## Introduction

The foremost common magnetic resonance imaging (MRI) markers of cerebral small vessel disease (SVD) are white matter hyperintensities (WMH)^1,2^. WMH are seen as hyperintense signal on T2-weighted or fluid-attenuated inversion recovery (FLAIR) and are surrounded by normal-appearing white matter (NAWM)^3^. Increasing evidence has shown that the conversion of NAWM into WMH is a continuous process^4,5^. Therefore, the visualization of gradual FLAIR signal changes could help bridge the gap between white matter and its heterogeneous pathology. Additionally, recent studies have shown pathophysiological changes in NAWM adjacent to WMH, including neuroinflammation and lower cerebral blood flow (CBF)^6–8^, suggesting that even though MRI is the most widely used tool to investigate the etiology of WMH, current imaging and its dichotomous analysis does not adequately capture the underlying pathology.

Vascular inflammation is increasingly suggested to be a major contributor to the transition from NAWM to WMH in individuals with hypertension^6^. Unfortunately, the interplay between vascular inflammation and the vasculature itself remains poorly understood since pathological investigation of the complex human cerebrovascular network (and its direct environment) three-dimensionally in both WMH and NAWM was lacking. Pathological studies of *post-mortem* brains have shown that microvascular rarefaction and neuroinflammation underlie WMH^9–11^. However, human SVD (immuno-)histopathological evidence has been limited to small two-dimensional field of views, often restricted to very thin tissue sections (few micrometers), which might overlook the interaction between the vast vascular network and neurovascular inflammation occurring in the brain parenchyma. Kugler et al*.*, recently published an open-source tool suitable for automated and robust three-dimensional cerebrovascular analysis^12^, which application could help to elucidate unprecedented vascular, structural and neurovascular inflammatory changes in NAWM and WMH. Hence, pathological studies using novel immunolabeling and analysis techniques for accurate characterization of the microvasculature and neurovascular inflammation are necessary to elucidate their interplay in the progression of WMH.

In this case report, we examined 3D vascular and neurovascular inflammatory alterations on over 10^3^ fold volumetric dataset compared to standard two-dimensional histology using state-of-the-art light sheet fluorescence microscopy (LSFM)^13^ together with a modified iDISCO+ protocol^14^ to successfully clear human brain tissue of a patient with progression of WMH over a 7-year course. Moreover, we automatically segmented both WMH and NAWM using a deep learning-based model, which was also able to (sub-)segment these into additional regions of interest based on FLAIR signal to examine whether there is a relation between MRI intensity variations and the underlying microvascular pathology and neurovascular inflammation visible on LSFM. The results provide a better understanding of microvascular pathology and neurovascular inflammation underlying NAWM and WMH, and may help to develop potential therapeutic targets for early stages of SVD.

## Methods

### Case presentation

**Figure 1:**
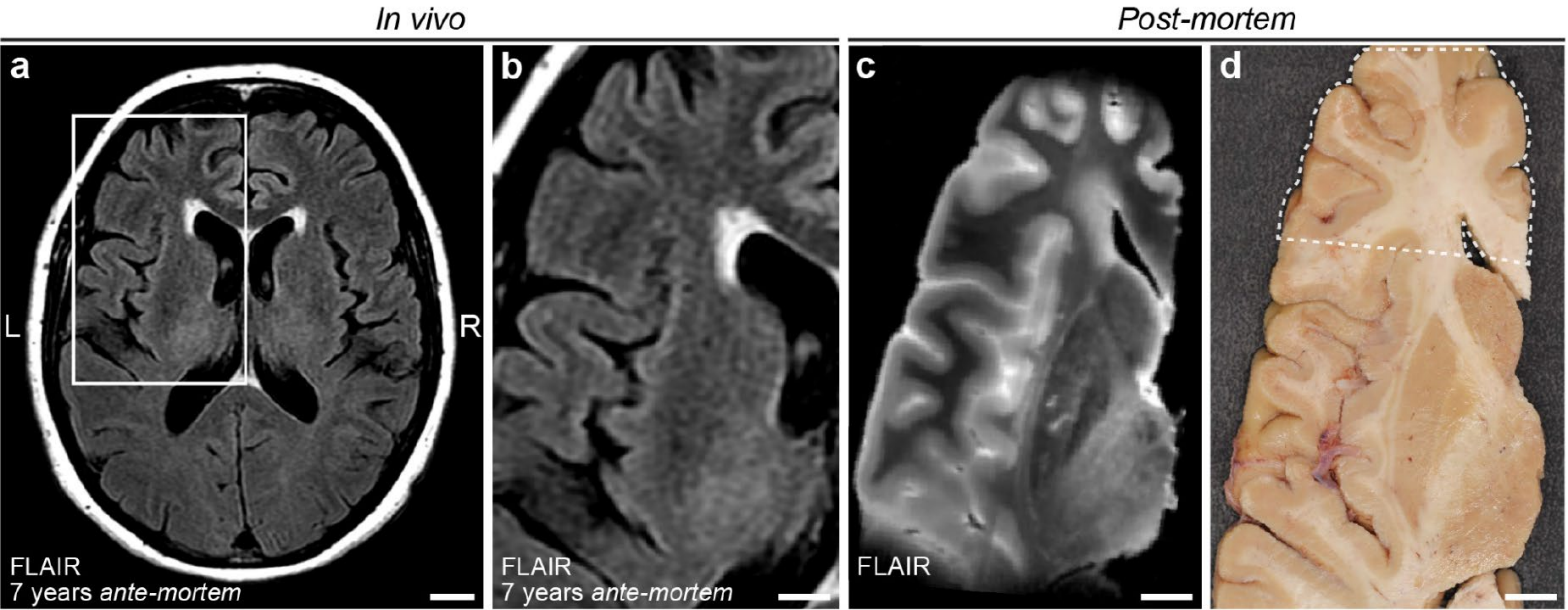
In vivo and post-mortem MRI. (**a**) In vivo fluid-attenuated inversion recovery (FLAIR) MRI (1.5 Tesla) axial view acquired 7 years prior to death, (**b**) close up of in vivo MRI showing the region of interest used for post-mortem MRI. (**c**) Post-mortem FLAIR MRI (7 Tesla) of left hemisphere. (**d**) Photograph of the slab sectioned from the left hemisphere that was used for tissue immunolabeling and clearing. Dashed outline illustrates the exact region of interest. Scale bars: 1 cm (**a–d**).

We investigated a 73-year-old female with a medical history of chronic hypertension, obesity, steatosis, chronic obstructive pulmonary disease (COPD) and smoking, who had consented to participate in our brain donation program. This individual showed no signs or symptoms of inflammatory, autoimmune or demyelinating diseases in her medical history. Cause of death was lung cancer, with no clinical and radiological signs of cerebral metastasis. A 1.5 T MRI scan had been made for diagnostic reasons seven years before her death and showed mild WMH (Fazekas grade 1; Fig. 1a,b)^15^. Severe WMH were observed in *post-mortem* MRI examination (Fig. 1c). Additionally, we performed LSFM in blocks of the *post-mortem* human brain tissue (Fig. 1d) with a modified iDISCO+ protocol^14^ in order to characterize three-dimensionally vascular, neuro- and (vascular) inflammatory changes linked to white matter pathology throughout a broad brain region. All protocols concerning data acquisition and tissue processing were approved by the Medical Ethics Review Committee (Commissie Mensgebonden Onderzoek (CMO) region Arnhem-Nijmegen, The Netherlands, file No. 2017-3941), and are legislated under Dutch national law (BWBR0005009).

### MRI acquisition

In vivo MRI was acquired on a Signa Excite 1.5 Tesla MRI Scanner (General Electric Company, Boston, MA, USA) 7 years before death.

Before and after fixation, the left hemisphere was scanned *post-mortem* at room temperature on a Bruker 7 Tesla Clinscan MR system (Bruker Biospin, Ettlingen, Germany) interfaced with a Siemens Syngo VB15 console. The brain was fixed with phosphate-buffered 4% paraformaldehyde (PFA) for a period of 4 months before the second *post-mortem* scan. Both scans were acquired with a FLAIR sequence at a resolution of 500 × 500 × 500 µm (TR = 8200 ms, TE = 39 ms, TI = 1600 ms, 2 averages). Further details on *post-mortem* MRI data acquisition are described elsewhere^6^.

### Deep learning model for post-mortem MRI data segmentation

In order to examine FLAIR MRI, we used a deep learning-based model to segment WMH, NAWM and grey matter (GM) as visualized on *post-mortem* MRI (code is available on GitHub (https://github.com/MatthijsLuijten/WMH_tool/tree/main/WMH_new)). This model was trained on annotations (ground truth) made by BG and GSG on the larger *post-mortem* MRI dataset of individuals with and without hypertension^6^. The model consisted of ensembled fully convolutional networks in the form of U-Nets^16^, including a final ‘layer’ that mapped the feature vectors to four prediction classes: WMH, NAWM, GM, and background. Each U-Net model generated a probability prediction map, which were averaged and transformed into a segmentation map encompassing all predictions. To enhance training, we utilized both annotated and augmented data generated with the aid of Albumentations, a Python library^17^.

**Figure 2:**
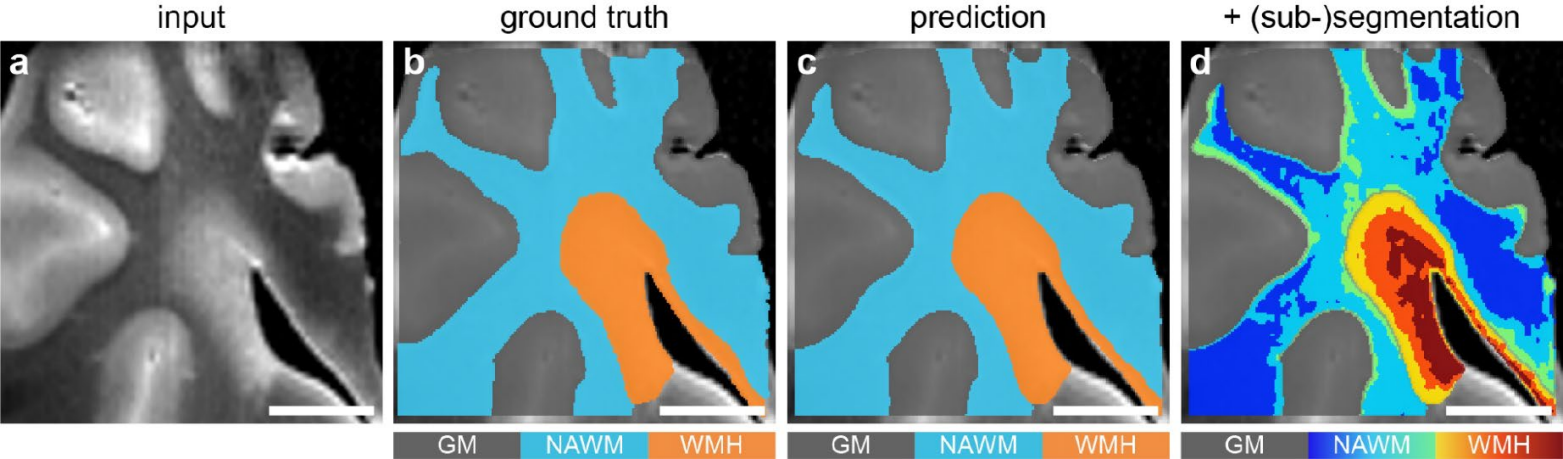
Overview of segmentation pipeline. (**a**) Post-mortem high-field fluid-attenuated inversion recovery (FLAIR) MRI corresponding to the tissue slab used for light sheet fluorescence microscopy. (**b**) This image illustrates the manual segmentation result as ground truth. (**c**) This image shows the segmentation outcome known as prediction for grey matter (GM), normal-appearing white matter (NAWM) and white matter hyperintensity (WMH). (**d**) After successful segmentation of grey and white matter, we applied KMeans clustering algorithm to (sub-)segment both NAWM and WMH based on changes on FLAIR signal. Scale bars, 1 cm (**a–d**).

After successful segmentation into the aforementioned predictions, KMeans clustering algorithm^18^ was used on normalized FLAIR (input) to classify WMH and NAWM into distinct regions of interest based on MRI-visible changes. Briefly, FLAIR signal values were used as features to generate (sub-)segmentation classes within NAWM and WMH, where distance was also used as a measure of similarity. An example of the (sub-)segmentation output can be seen in Fig. 2.

### Light sheet tissue preparation and immunostaining

**Figure 3:**
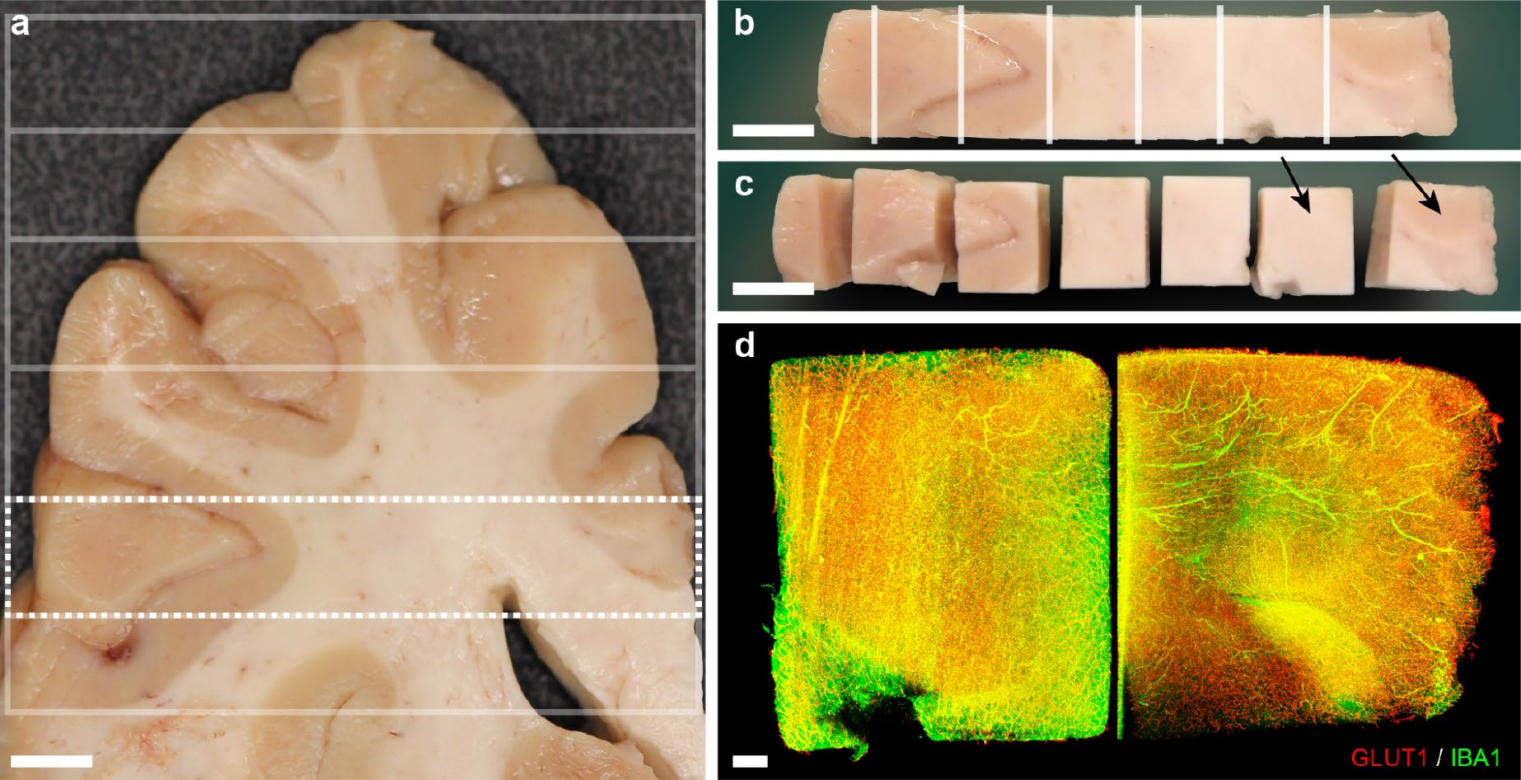
Cubes preparation for immunolabeling and clearing protocol. (**a**) Axial tissue slab corresponding to the region of interest used for immunolabeling and clearing. The white grid illustrates how the slab was initially cut into several rows. Dashed rectangle displays tissue blocks in (**b**). White lines in (**b**) show the section areas to produce the cubes shown in (**c**). The black arrows in (**c**) point to the cubes shown in (**d**) after immunolabeling for both glucose transporter 1 (GLUT1) and ionized calcium-binding adapter molecule 1 (IBA1), clearing and imaging. Scale bars: 0.5 cm (**a–c**), 500 µm (**d**).

After MRI acquisition, the ventral part of the left hemisphere was divided into an axial slab of ~ 6 mm of thickness (Fig. 1d). For LSFM staining, the axial slab was divided in cubes of approximately 0.125 cm^3^ from the most anterior part of the frontal lobe to the anterior part of the caudate nucleus head (Fig. 3, Supplementary Fig. S1). The tissue cubes were handled according to the iDISCO+ protocol^14^, with some modifications. Cubes were immunolabelled for glucose transporter 1 (GLUT1) to visualize blood vessels^19^ and subsequently measure vascular integrity based on vascular network architecture, and for ionized calcium-binding adapter molecule 1 (IBA1) to detect activated macrophages and microglia (Fig. 3d). The full protocol is listed in the Supplementary Information, modifications to the protocol are listed here. The samples were incubated in the permeabilization solution for 4 days, with the primary antibodies (rabbit anti-GLUT1 (07-1401; Millipore, Burlington, MA, USA; 1:525; RRID: AB_11212210) and goat anti-IBA1 (ab5076; Abcam, Cambridge, UK; 1:400; RRID: AB_2224402)) for 3 weeks, and with the secondary antibodies (donkey anti-rabbit Alexa Fluor Plus 647 (A32795; Thermo Scientific, Waltham, MA, USA; 1:200; RRID: AB_2762835) and donkey anti-goat Alexa Fluor Plus 555 (A32816; Thermo Scientific, Waltham, MA, USA; 1:200; RRID: AB_2762839)) for 3 weeks. The dehydrations steps before clearing were extended to 2 h per step and the samples were incubated in 100% methanol for 48 h instead of overnight. Delipidation steps were extended to 1 × 20 min and 1 × 40 min.

### Light sheet imaging

Samples were imaged using a light sheet fluorescence microscope (Ultramicroscope II, LaVision Biotec, Bielefeld, Germany) equipped with an sCMOS camera (Andor Neo) and a × 1.1 objective (effective magnification × 2.2, NA 0,1, LaVision Biotec), NTK Photonics white-light laser and filter sets for 555 nm and 647 nm excitation. Imaging was performed using ImSpector Pro (version 7.1.4; LaVision Biotec) with a resolution of 2.95/2.95/3 µm in x/y/z (3 µm z-steps), the “dynamic focus” option (15–20 steps depending on cube size), a single light sheet coming from left and right, and 100 ms camera exposure time.

### Light sheet data processing and analysis

All cubes were pre-processed using Arivis Vision 4 dimensional (4D) (version 3.6.2; Arivis AG, Rostock, Germany) background correction module with defined threshold per channel. 3D reconstruction of the brain region from the cleared cubes was completed using Arivis Vision 4D volume fusion module. Cubes were fused to each other based on macroscopical landmarks such as grey and white matter distribution, large blood vessels, etc. documented prior to clearing. First, cubes were fused by rows, based on their neighboring cubes, and thereafter each row was fused to their parallel rows (Fig. 3). Cubes were excluded from volume fusion due to fragmentation/small size (4/34), shrinkage (1/34), major grey matter composition (4/34) and low signal-to-noise ratio (3/34).

Six cubes were selected for analysis based on their anatomical position, from WMH towards NAWM (Supplementary Fig. S1). Raw 16-bit images (GLUT1, IBA1) were loaded into ImageJ (version 1.53t, National Institute of Health, Bethesda, MD, United States) and converted to 8-bit. In order to correlate MRI-LSFM, the resulting 8-bit images were divided into 0.16 mm^2^ tiles corresponding to xy dimensions of MRI voxels.

#### Assessment of microvascular pathology

**Figure 4:**
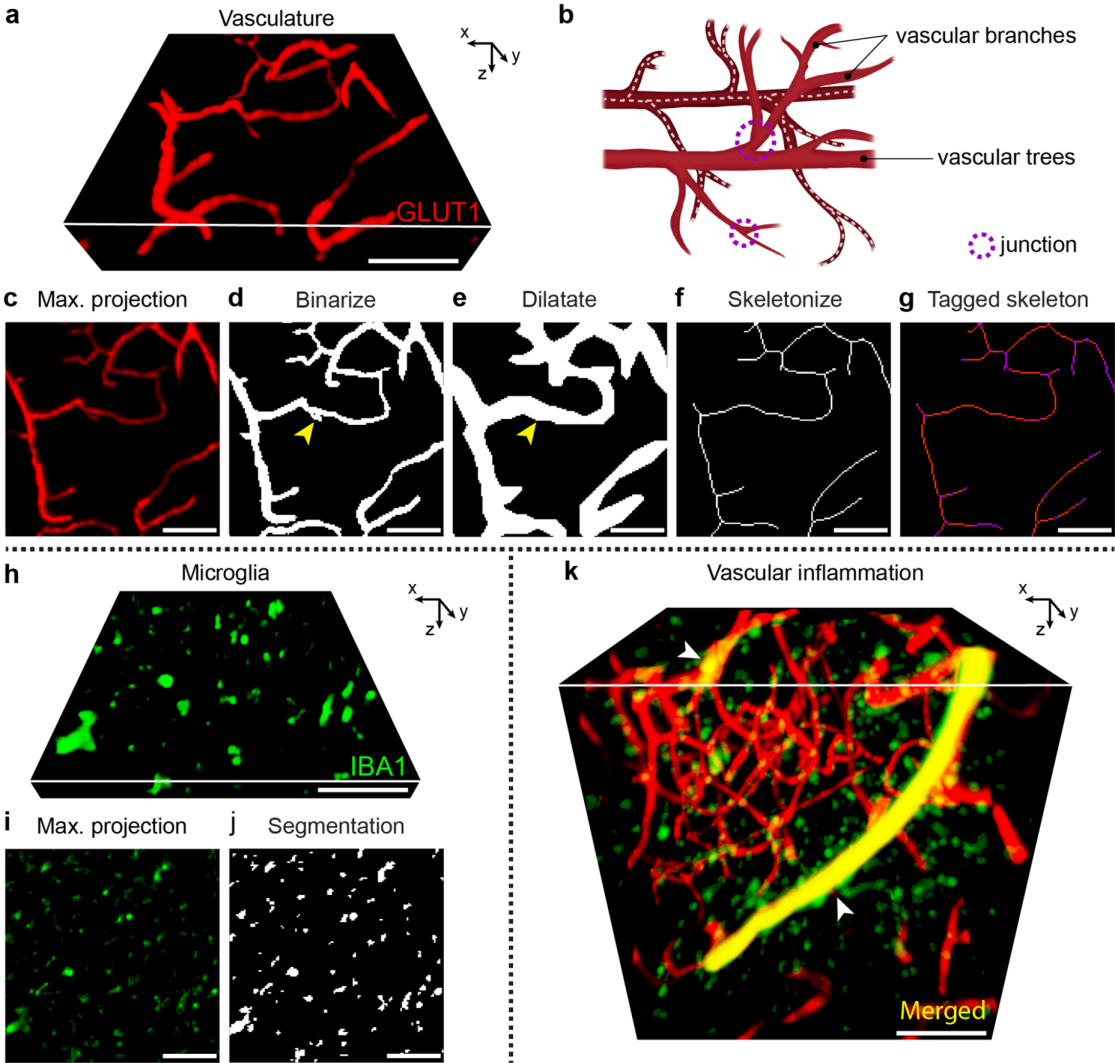
Overview of vascular network and neurovascular inflammation processing and analysis pipelines. (**a**) Three-dimensional 90-µm sub-stack of light sheet fluorescence microscopy of glucose transporter 1 (GLUT1). (**b**) Schematic representation of vascular network analysis parameters included. Vascular network length is depicted as dotted line following a vascular tree since it corresponds to the total vascular network course. Junctions are points where the vascular network bifurcates. (**c**) Maximum projection obtained from the sub-stack shown in (**a**). Images were later (**d**) binarized and (**e**) dilatated. The dilatation step prevents very small branches (yellow arrowhead) visible on the binary image as adjacent from beginning to end to be misclassified as round junctional sections. (**f**) Skeletonized and (**g**) tagged topological vascular network (branches are shown in red, junctions in purple). The visible junctions correspond to both two-dimensional and three-dimensional junction sections. (**h**) Three-dimensional 30-µm sub-stack of light sheet fluorescence microscopy of ionized calcium-binding adaptor molecule 1 (IBA1). (**i**) Maximum projection obtained from the sub-stack shown in (**h**). (**j**) Microglia refined segmentation output by cell size [9–900 µm^2^] and circularity [0.2–1.0]. (**k**) Three-dimensional exemplification of vascular inflammation (yellow; white arrowheads) of three-dimensional light sheet fluorescence microscopy of GLUT1 (red) together with IBA1 (green). Scale bars: 100 µm (**a,c–k**).

For GLUT1 (Fig. 4a–g), enhancement of the vascular staining was performed using Sato enhancement filter^20^ in ImageJ based on the zebrafish vasculature quantification (ZVQ) pipeline^12^. In order to accurately recapitulate the complex architectural network of the vasculature (depicted schematically in Fig. 4b) we generated maximum intensity projections from 90 µm thick sub-stacks (Fig. 4a,c). Enhanced images were segmented to isolate the target staining from background, binarized (Fig. 4d) and processed through skeletonization^21^ to extract their topological vascular skeleton (Fig. 4e–g). The resulting topological vascular skeleton was analyzed using the Analyze skeleton plug-in in ImageJ^22^, which provided novel measures for total vascular network length, mean vascular branch length, number of vascular trees and branches per cubic millimeter in the context of SVD. To evaluate vascular tree structure complexity, the variable branches per tree was computed as the ratio between the average number of branches and trees. Vascular tortuosity index was calculated by diving each branch length by its respective Euclidian distance, shortest distance between start and end of the vascular branch, and thus values above 1 indicate increased tortuosity of the vascular branch. Finally, vascular volume was automatically calculated in ImageJ.

#### Evaluation of neuro- and vascular inflammation

For IBA1 (Fig. 4h–j), raw images were smoothed using a 3D Median kernel (x/y/z (pixel):1.02/1.02/1). Microglia cells were segmented from the background and filtered for noise by targeting pixels above 9 µm^2^ surface. Maximum intensity projections were generated from 30 µm sub-stacks (Fig. 4h,i). Microglia segmentation was refined by size [9–900 µm^2^] based on evidence from a high magnification confocal imaging study^23^. Additionally, based on our previous findings we refined microglia segmentation by circularity [0.2–1.0]^6^ to exclusively measure scattered microglial cells (Fig. 4j) opposed to those adjacent to blood vessels or clustered throughout the cubes. Frequency of microglial cells IBA1 was automatically counted by ImageJ (number per mm^3^). Next, scattered, clustered [≥ 900 µm^2^] and total microglial cells volume was automatically calculated by ImageJ.

Finally, vascular inflammation (Fig. 4k) was automatically calculated in ImageJ by calculating the colocalization (overlapping volume) between GLUT1+ vasculature and microglia over the total vascular volume.

### Statistics

**Figure 5:**
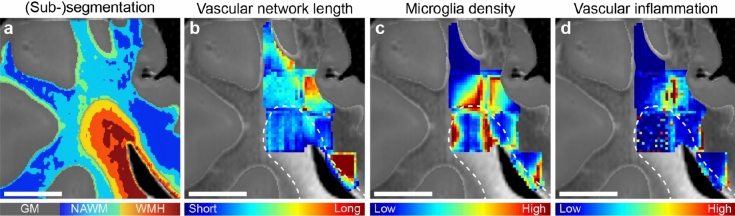
Vascular network length, microglia density and vascular inflammation in WMH and NAWM. (**a**) (Sub-)segmentation of both normal-appearing white matter (NAWM) and white matter hyperintensity (WMH) based on changes on fluid-attenuated inversion recovery (FLAIR) signal. (**b**) Representative voxel-wise image of vascular network length (*GLUT1* glucose transporter 1) analyzed across tissue blocks immunolabeled and cleared with a modified iDISCO+ protocol. In WMH (white dashed line; n = 294), vascular network length was approximately 30% shorter compared to NAWM (p < 0.001; n = 664). Vascular network length showed a negative correlation with WMH (sub-)segmentation (ρ =  − 0.272; p < 0.001), suggesting underlying vascular network architecture was shorter with increasing MRI signal. (**c**) Representative voxel-wise image of microglia density (*IBA1* ionized calcium-binding adaptor molecule 1). The amount of microglia per mm^3^ in WMH (white dashed line) was larger than NAWM (p < 0.001). (**d**) Representative voxel-wise image of vascular inflammation (colocalization between IBA1 and GLUT1). Colocalization analysis showed that vascular inflammation was larger in NAWM than WMH (p < 0.001). Min–max normalization was used for the data shown in (**b–d**). *GM* grey matter, scale bars, 1 cm (**a–d**).

Statistical analysis of the data was performed using IBM SPSS statistics 27 (IBM Corporation, Armonk, NY, USA). Medians and interquartile ranges (IQR) were calculated for all variables. All variables were tested for outliers and normality. Due to significant deviations from normal distribution, differences between WMH and NAWM variables were analyzed using a non-parametric Kruskal–Wallis test. Spearman correlation coefficients were used to analyze the correlation among vascular and neurovascular inflammation changes and FLAIR signal (sub-)segmentation classes depicted in Fig. 5a. The Bonferroni correction method was applied to account for multiple comparisons in the statistical analysis. The desired significance level (α) of 0.05 was adjusted by dividing it by the number of total comparisons (n = 13), resulting in a Bonferroni corrected significance level of α/13 = 0.004 (two-tailed). p-values less than or equal to 0.004 were considered statistically significant.

## Results

### Microvascular pathology in 3D of WMH and NAWM

We observed that the total vascular network was ~ 30% shorter in length in WMH compared to NAWM (p < 0.001; Table 1, Fig. 5b, Supplementary Movie S1). However, there were no differences in vascular volume between regions. Average branch length (p < 0.001) was larger in WMH than NAWM. While there were no significant changes in mean vascular branches—vessel bifurcation—per cerebrovascular tree network, overall density of cerebrovascular trees (p < 0.001) and branches per mm^3^ (p < 0.001) was lower in WMH than NAWM. We did not observe differences regarding vascular tortuosity index in WMH and NAWM.

**Table 1:** Vascular and neurovascular inflammation by regions of interest.

	NAWM Median (IQR)	WMH Median (IQR)	p value
			WMH vs NAWM
Vasculature (GLUT1)			
Vascular volume (mm^3^)	2.3 × 10^–5^ (1.7 × 10^–5^–8.4 × 10^–5^)	2.8 × 10^–5^ (1.7 × 10^–5^–3.7 × 10^–5^)	0.958
Network length (mm)	48.3 (36.0–70.5)	33.4 (27.1–41.2)	** < 0.001**
Average branch length (µm)	2.6 (1.6–3.8)	6.9 (3.7–9.2)	** < 0.001**
Density of vascular trees (#/mm^3^)	294.4 (167.4–1183.1)	197.3 (154.6–335.1)	** < 0.001**
Density of vascular branches (#/mm^3^)	1412.5 (1085.6–2180.6)	874.0 (745.5–1014.8)	** < 0.001**
Branching per tree^a^	5.1 (1.3–8.3)	4.6 (2.3–6.5)	0.086
Vascular tortuosity index (≥ 1)	1.20 (1.18–1.25)	1.22 (1.19–1.26)	0.007
Microglia (IBA1)			
Microglia density (#/mm^3^)	3809.8 (799.6–7293.4)	4275.0 (2686.2–7919.4)	** < 0.001**
Scattered microglia volume (mm^3^)	0.008 (0.001–0.020)	0.008 (0.005–0.016)	0.060
Clustered microglia volume (mm^3^)	0.003 (0.0001–0.019)	0.003 (0.0009–0.008)	0.006
Total microglia volume (mm^3^)	0.012 (0.002–0.042)	0.011 (0.006–0.026)	0.878
Vascular inflammation (GLUT1 and IBA1)			
Vascular inflammation^b^ (%)	2.2 (0.4–6.5)	1.6 (0–3.9)	** < 0.001**

*GLUT1* glucose transporter 1, *IBA1* ionized calcium-binding adaptor molecule 1, *IQR* interquartile range (IQ25–IQ75), *NAWM* normal-appearing white matter, *WMH* white matter hyperintensity.

^a^Number of vascular bifurcations (branches) per vascular tree network.

^b^Colocalization between microglia and vasculature.

Significant values are in bold.

### Neuro- and vascular inflammation in 3D of WMH and NAWM

The number of microglia per mm^3^ (p < 0.001; Table 1, Fig. 5c) was 30% higher in WMH compared to NAWM. However, we did not observe differences in scattered, clustered or overall microglial volume between regions. Assessment of vascular inflammation—colocalization of microglia and vasculature—showed that in NAWM vascular inflammation was higher compared to WMH (p < 0.001; Table 1, Fig. 5d, Supplementary Movie S1).

### Beyond white matter dichotomy: examining 7 T MRI signal

We measured the relation between stratified FLAIR signal within white matter—from normal-appearing to most hyperintense—and vascular network and neurovascular inflammation. Across all white matter regions, total vascular network length (r =  − 0.240, p < 0.001; Table 2), vascular inflammation (r =  − 0.192, p < 0.001), density of cerebrovascular trees (r =  − 0.167, p < 0.001) and branches per mm^3^ (r =  − 0.375, p < 0.001) were negatively associated with FLAIR signal. Total vascular volume (r = 0.116, p < 0.001), amount of microglia per mm^3^ (r = 0.109, p = 0.018) and average branch length (r = 0.443, p < 0.001) were positively correlated with FLAIR signal.

**Table 2:** Vascular and neurovascular inflammation from normal-to-hyperintense white matter.

	All (normal-to-hyperintense)	NAWM	WMH
	ρ (p value)	ρ (p value)	ρ (p value)
Vasculature (GLUT1)			
Vascular volume	**0.116 (p < 0.001)**	**0.267 (p < 0.001)**	− 0.079 (p = 0.212)
Network length	− **0.240 (p < 0.001)**	− 0.010 (p = 0.798)	− **0.272 (p < 0.001)**
Average branch length	**0.443 (p < 0.001)**	− 0.002 (p = 0.970)	**0.266 (p < 0.001)**
Density of vascular trees per mm^3^	− **0.167 (p < 0.001)**	0.009 (p = 0.818)	− 0.017 (p = 0.787)
Density of vascular branches per mm^3^	− **0.375 (p < 0.001)**	0.047 (p = 0.248)	− **0.332 (p < 0.001)**
Branching per tree	− 0.006 (p = 0.862)	− 0.016 (p = 0.696)	− 0.167 (p = 0.008)
Vascular tortuosity index (≥ 1)	− 0.020 (p = 0.563)	− **0.239 (p < 0.001)**	− 0.015 (p = 0.819)
Microglia (IBA1)			
Microglia density per mm^3^	**0.109 (p = 0.001)**	− 0.032 (p = 0.424)	− 0.068 (p = 0.284)
Scattered microglia volume	0.014 (p = 0.677)	− 0.058 (p = 0.151)	− 0.161 (p = 0.011)
Clustered microglia volume	− 0.069 (p = 0.043)	− **0.129 (p = 0.001)**	− **0.252 (p < 0.001)**
Total microglia volume	− 0.026 (p = 0.438)	− 0.097 (p = 0.016)	− **0.208 (p < 0.001)**
Vascular inflammation (GLUT1 and IBA1)			
Vascular inflammation	− **0.192 (p < 0.001)**	− 0.107 (p = 0.008)	− 0.100 (p = 0.117)

*GLUT1* glucose transporter 1, *IBA1* ionized calcium-binding adaptor molecule 1, *IQR* interquartile range (IQ25–IQ75), *NAWM* normal-appearing white matter, *WMH* white matter hyperintensity.

Significant values are in bold.

Similarly, in WMH total vascular network length (r =  − 0.272, p < 0.001) and density of vascular branches per mm^3^ (r =  − 0.332, p < 0.001) showed a negative association with FLAIR signal. Additionally, volume of clustered microglial cells (r =  − 0.252, p < 0.001) and volume of total microglial cells (r =  − 0.208, p < 0.001) showed a negative association with FLAIR signal solely in WMH. Average branch length (r = 0.266, p < 0.001) was positively correlated with FLAIR signal.

In NAWM, we observed that vascular tortuosity index (r =  − 0.239, p < 0.001) and volume of clustered microglial cells (r =  − 0.129, p = 0.001) negatively correlated with FLAIR signal. On the other hand, total vascular volume (r = 0.267, p < 0.001) positively correlated with FLAIR signal. Altogether these findings suggest that both vascular and inflammatory changes already occur within NAWM itself, and that these correlate with subtle FLAIR signal alterations.

## Discussion

In this MRI-LSFM case study on the underlying 3D pathology of WMH, and adjacent NAWM, we found that the vascular network in WMH was not only shorter, but also less intricate compared to NAWM. Even though we observed a larger density of microglia in WMH than NAWM, vascular inflammation was notably higher in NAWM compared to WMH. Additionally, we examined gradual changes in FLAIR signal and their correlation to 3D vascular and neurovascular inflammation, and we observed different degrees of vascular pathology in both NAWM and WMH beyond the standard white matter dichotomy. These differences might explain the large heterogeneity previously linked to WMH, highlighting the need to understand underlying MRI signal changes to evaluate WMH severity in patients.

Microvascular rarefaction in WMH^9,24^ and decreased CBF^7,8^ have been linked to the origin of WMH^25–27^. However, 3D pathological study of the intricate human cerebrovascular network, including its immediate surroundings, in both WMH and NAWM, was still lacking. Therefore, we investigated microvascular and neurovascular inflammatory changes in 3D to get a deeper insight on white matter’s underlying pathology. We found that in WMH the vascular network, visualized by GLUT1 staining, was shorter and showed a lower number of bifurcations compared with NAWM. Our findings are in line with the well-established fact that microvascular rarefaction is common in elderly^28^, however, our 3D examination showed that microvascular pathology extended throughout the vast vascular network architecture in white matter, including the NAWM. Notably, the negative correlation between both vascular network length and density of vascular branches with the degree of MRI signal within WMH highlights the heterogeneity of microvascular pathology underlying WMH. These findings, in line with Phuah et al.^29^, demonstrate the variability of microvascular pathology extent within WMH itself. It is possible that in the lesion core—higher FLAIR signal—recovery mechanisms such as hypoxia-induced angiogenesis^28^ are impaired, and thereafter the pathological cascade could be shifted towards inflammation and neurodegeneration, causing further tissue damage^30,31^. Contrarily, regions surrounding the lesion core that are also bright on FLAIR, but not as hyperintense, might still have functional recovery mechanisms to compensate for ischemic injury^32^, possibly explaining the occasional occurring phenomenon known as WMH regression^33^. As our study deals with detailed analysis of one individual, future research should include more individuals with different degrees of WMH burden and other vascular, inflammatory and neurodegenerative markers to understand the relevance of MRI (e.g., FLAIR, T2) signal differences within white matter, and particularly in WMH.

We found that the vascular tortuosity index was not different between WMH and NAWM. The individual included in our study was of advanced age, a factor that has been previously linked to increased tortuosity^34^. Furthermore, previous examination of vascular tortuosity showed that the most tortuous vessels were found in the external- and extreme capsules, as well as in subcortical region of the insula^35^. Suggesting that vascular tortuosity might be linked to the physical properties of white matter^35^. Altogether, our results seem to indicate that vascular tortuosity (> 1.25) might be a common finding in deep white matter regions among the elderly, as observed in the blood vessels analyzed in both WMH and NAWM.

Previous studies have shown increased microglial activation underlying WMH^10,11,36^ and, to a lesser extent, signs of neuroinflammation have been recently found in NAWM^6^. Likewise, our results showed increased microglial activation in WMH. Furthermore, examination of vascular inflammation/endothelial dysfunction on serum markers, such as homocysteine, has shown a robust association between SVD burden and vascular inflammation (reviewed by^37^), implying that microvascular pathology plays a major role in the early pathology of SVD. Remarkably, we found that vascular inflammation was larger in NAWM than WMH. Although we cannot infer causality, the fact that neuroinflammation remained larger in WMH suggests that vascular inflammation might be critical in converting NAWM areas to WMH. Environmental stressors, including hypertension, are known to induce microvascular dysfunction, which in turn can promote vascular inflammation^38^. As a result, altered vascular permeability associated with microvascular dysfunction could exacerbate inflammation and microvascular damage^38^. Therefore, it is possible that neurovascular inflammation may significantly contribute to WMH progression by furthering microvascular dysfunction, and ultimately microvascular rarefaction^6,39^. This emphasizes the need to further the current understanding on vascular inflammation and its link to SVD etiology. Hence, more studies combining vascular and inflammatory markers such as matrix metallopeptidase 9 (MMP-9) are needed to fully understand the relationship between vascular inflammation and microvascular rarefaction.

In order to better understand white matter as seen on MRI, we examined vascular network pathology and neurovascular inflammation across intensity-stratified regions of interest. Interestingly, increasing signal as detected on MRI (in both WMH and NAWM) displayed a positive correlation with vascular volume, with this association being stronger in NAWM. Taken together with the fact that MRI signal across white matter positively correlated with neuroinflammation, these findings suggest that MRI is sensitive enough to detect subtle pathological changes. Therefore, further research is needed to explore the underlying mechanisms to improve our understanding on MRI-visible white matter pathology.

Some limitations of our study should be considered. Unfortunately, the long interval between in vivo and *post-mortem* MRI prevented the comparison between the two timepoints and white matter underlying pathology. Scanning intervals above 3 years increase the chances of WMH progression, and thereafter hinder the link to the pathology visible on *post-mortem* examination^40^. Consequently, we solely examined MRI-LSFM correlations on *post-mortem* MRI. Secondly, MRI-LSFM associations have been limited to WMH, and its adjacent NAWM, due to the specimen size restriction linked to the optical clearing protocol. While we cleared and immunolabeled many tissue cubes to get an accurate overview of white matter changes from WMH into NAWM, this resulted in a very extensive protocol. Consequently, our 3D pathological examination was limited to one individual. Nonetheless, we were able to visualize underlying white matter pathology on over 10^3^ fold volumetric dataset compared to standard two-dimensional histology. Additionally, our findings are in line with prior neuropathological studies^6,9–11,24,36^, but furthering the understanding of vascular changes throughout an extensive characterization of 3D vascular network architecture highlighting for the first time the structural vascular changes, including length, ramifications, tortuosity, as well as the extent of neurovascular inflammation across white matter in detail. Additionally, we observed that vascular inflammation was larger in NAWM. These findings not only reinforce the hypothesis that neuroinflammation is an important risk factor in the context of SVD, but also imply that vascular inflammation plays a major role at the earliest stages towards WMH progression in those with hypertension. Additionally, this is the first comprehensive three-dimensional study of vascular pathology underlying WMH using deep learning-based automatic (sub-)segmentation of FLAIR signal. Our results showed that FLAIR signal could already reflect vascular alterations in NAWM and WMH, suggesting that FLAIR signal in WMH might not solely correlate with myelin and axonal loss, and thereafter additional pathological studies of white matter (sub-)segmentation could elucidate the most predominant pathologies behind FLAIR signal alterations, which might precede the conversion to WMH.

In conclusion, higher vascular inflammation in NAWM compared to WMH provided further evidence for a role of vascular inflammation in the pathogenesis of WMH. FLAIR signal correlated differently to varying degrees of vascular pathology not only in NAWM, but also in WMH. These findings emphasize the need to elucidate the underlying mechanisms of MRI-visible alterations already occurring in NAWM, not only in WMH. Further examination of neurovascular inflammation across MRI-visible alterations could aid deepening our understanding on WMH conversion, and thereafter how to improve the prognosis of WMH burden in the context of SVD.

### Ethics approval and consent to participate

The individual included in this case report signed informed consent to use their medical records for research purposes, publication, autopsy and use of tissue. All protocols concerning data acquisition and tissue processing were approved by the Medical Ethics Review Committee (Commissie Mensgebonden Onderzoek (CMO) region Arnhem-Nijmegen, The Netherlands, file No. 2017-3941), and are legislated under Dutch national law (BWBR0005009).

### Supplementary Information

Supplementary Information.

Supplementary Legends.

Supplementary Movie S1.

## Data Availability

The datasets generated and/or analyzed during the current study are available upon reasonable request to the corresponding author.
