# Supplementary material for: Three-dimensional identification of microvascular pathology and neurovascular inflammation in severe white matter hyperintensity: a case report
Source: Sci Rep. 2024 Feb 29;14:5004. doi: 10.1038/s41598-024-55733-y (PMC10904845; doi:10.1038/s41598-024-55733-y)
Supplement: Supplementary file 1 — Supplementary Information. [file 41598_2024_55733_MOESM1_ESM.pdf]

Supplementary information

Light sheet immunostaining protocol

All steps at room temperature (RT) were done on a roller mixer, and all steps at 37°C were exerted on a rotating platform.

First, the cubes were dehydrated using an increasing concentration of methanol (MeOH) (20%, 40%, 60%, 80%, 2x 100%) in dH2O at RT, for 1 hour per concentration step. Then they were washed overnight in 66% dichloromethane (DCM; 270997; Sigma-Aldrich, St Luis, MO, USA)/33% MeOH and the next day in pure MeOH for 2x 4 hours. Thereafter the cubes were put at 4°C for 30 minutes and then transferred to a 5% H2O2 in MeOH bleaching solution, in which they were incubated overnight at 4°C. The following day they were rehydrated in decreasing concentrations of MeOH (80%, 60%, 40%, 20%), phosphate-buffered saline (PBS), and 2x in PTx.2 (PBS with 0,2% Triton X-100). Then the cubes were incubated in permeabilization solution (20% Dimethyl sulfoxide (DMSO) and 0,3M glycine in PTx.2) for 4 days and in blocking solution (10% DMSO and 6% normal donkey serum (NDS) in PTx.2) for 2 days at 37°C. Cubes were incubated with the primary antibodies rabbit anti-GLUT1 (07-1401; Millipore, Burlington, MA, USA; 1:525; RRID: AB_11212210) and goat anti-IBA1 (ab5076; Abcam, Cambridge, UK; 1:400; RRID: AB_2224402) in PTwH (PBS with 0,2% Tween-20 and 10 µg/ml heparin) with 5% DMSO and 3% NDS for 3 weeks at 37°C. Samples were washed in PTwH 5 times (3x 1h, 1x 2h, 1x overnight) at RT and incubated with the secondary antibodies donkey anti-rabbit Alexa Fluor Plus 647 (A32795; Thermo Scientific, Waltham, MA, USA; 1:200; RRID: AB_2762835) and donkey anti-goat Alexa Fluor Plus 555 (A32816; Thermo Scientific, Waltham, MA, USA; 1:200; RRID: AB_2762839) in PTwH with 3% NDS for 3 weeks at 37°C. Thereafter another 5 washing steps (3x 1h, 1x 2h, 1x overnight) were performed at RT (from here the tubes were wrapped in aluminum foil to prevent bleaching of the fluorescent signals). The cubes were dehydrated in MeOH (20%, 40%, 60%, 80%) in dH2O at RT, for 2 hours each step, and 3x in 100% MeOH (1x overnight, 1x 4 hours, 1x overnight). Delipidation was done in 66% DCM/33% MeOH for 3 hours and washing in 100% DCM (1x 20 minutes, 1x 40 minutes) at RT. Lastly, the cubes were cleared in dibenzyl ether (DBE; 108014; Sigma-Aldrich, St Luis, MO, USA; no shaking) and stored at RT until imaging.

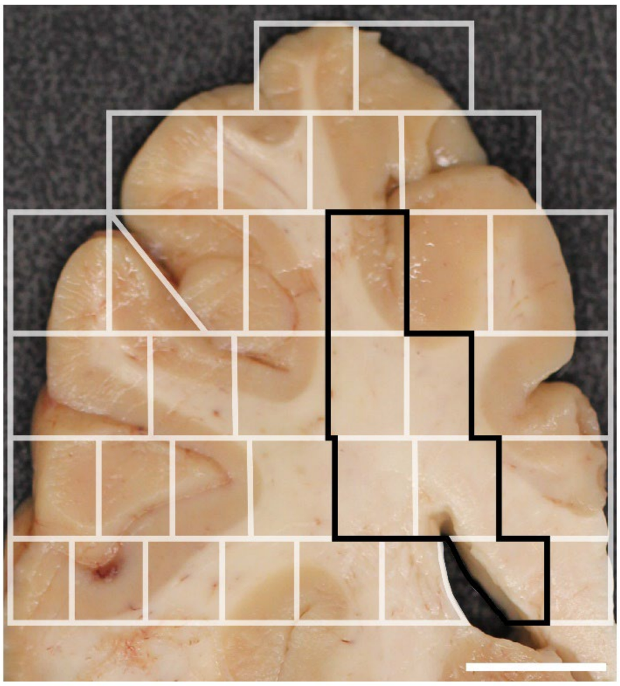

Supplementary Fig. S1: region of interest used for immunolabeling and clearing.

Photograph of axial tissue slab corresponding to the region of interest used for immunolabeling and clearing. The white grid illustrates the distribution of all blocks used for tissue immunolabeling and clearing. All blocks used for data analysis of microvascular pathology, neuroinflammation and vascular inflammation are included within the black irregular polygon (scale bar = 1 cm).
