# Supplementary material for: Three-dimensional identification of microvascular pathology and neurovascular inflammation in severe white matter hyperintensity: a case report
Source: Sci Rep. 2024 Feb 29;14:5004. doi: 10.1038/s41598-024-55733-y (PMC10904845; doi:10.1038/s41598-024-55733-y)
Supplement: Supplementary file 2 — Supplementary Legends. [file 41598_2024_55733_MOESM2_ESM.docx]

# Supplementary information description

ESM1.pdf

**Additional file 1: Immunolabeling and clearing protocol, and Fig. S1.** Full protocol for immunolabeling and clearing. The file also includes Figure S1, photograph of axial tissue slab corresponding to the region of interest used for immunolabeling and clearing (PDF 188 kb)

Supplementary movie S1.mov

**Additional file 2: Supplementary movie 1. From MRI to LSFM: a 3D visualization of microvascular pathology and neurovascular inflammation underlying white matter**

The movie starts with the visualization of white matter pathology as seen on 7Tesla MRI fluid-attenuated inversion recovery (FLAIR), zooming in on the region of interest used for light sheet fluorescent microscopy (LSFM). Within this region of interest, a white dashed line was used to point to the white matter hyperintensity (WMH). After, the movie shows the tissue blocks immunolabeled and cleared with a modified iDISCO+ protocol. Glucose transporter 1 (GLUT1) is shown in red and ionized calcium-binding adaptor molecule 1 (IBA1) is shown in green. The movie shows in yellow where both GLUT1 and IBA1 colocalize. The movie illustrates the microvascular rarefaction in WMH. Furthermore, the movie shows vascular inflammation in 3D within WMH and normal-appearing white matter (NAWM), both close and further from WMH (MOV 61456 kb)
